# Synthesis and Bioactivity Assessment of Novel Spiro Pyrazole-Oxindole Congeners Exhibiting Potent and Selective *in vitro* Anticancer Effects

**DOI:** 10.3390/molecules25051124

**Published:** 2020-03-03

**Authors:** Heba M. Abo-Salem, Amr Nassrallah, Ahmed A.F. Soliman, Manal S. Ebied, Mohamed E. Elawady, Sayeda A. Abdelhamid, Eslam R. El-Sawy, Yazeed A. Al-Sheikh, Mourad A. M. Aboul-Soud

**Affiliations:** 1 Chemistry of Natural Compounds Department, National Research Centre, Dokki, 12622 Giza, Egypt; hb_abosalem@yahoo.com (H.M.A.-S.); manalshaabanebied@gmail.com (M.S.E.); eslamelsawy@gmail.com (E.R.E.-S.); 2 Biochemistry Department, Faculty of Agriculture, Cairo University, 12613 Giza, Egypt; amotagly@cu.edu.eg; 3 Drug Bioassay-Cell Culture Laboratory, Pharmacognosy Department, National Research Center, Dokki, 12622 Giza, Egypt; ashehabeldin2007@yahoo.com; 4 Microbial Biotechnology Department, National Research Centre, Dokki, 12622 Giza, Egypt; mohamed_elawady82@yahoo.com (M.E.E.); sayeda.abdelrazek@yahoo.com (S.A.A.); 5 Chair of Medical and Molecular Genetics Research, Department of Clinical Laboratory Sciences, College of Applied Medical Sciences, King Saud University, P.O. Box 10219, Riyadh 11433, Saudi Arabia; yalsheikh@ksu.edu.sa

**Keywords:** isatin, spiro pyrazole-oxindoles, antiproliferative agents, apoptosis

## Abstract

The present work aims to design and synthesize novel series of spiro pyrazole-3,3’-oxindoles analogues and investigate their bioactivity as antioxidant and antimicrobial agents, as well as antiproliferative potency against selected human cancerous cell lines (i.e., breast, MCF-7; colon, HCT-116 and liver, HepG-2) relative to healthy noncancerous control skin fibroblast cells (BJ-1). The mechanism of their cytotoxic activity has been also examined by immunoassaying the levels of key anti- and proapoptotic protein markers. The analytical and spectral data of the all synthesized target congeners were compatible with their structures. Synthesized compounds showed diverse moderate to powerful antimicrobial and antioxidant activities. Results of MTT assay revealed that seven synthesized compounds (i.e., 11a, 11b, 12a, 12b, 13b, 13c and 13h) particularly exhibited significant cytotoxicity against the three cancerous cell lines under investigation. Ranges of IC_50_ values obtained were 5.7–21.3 and 5.8–37.4 µg/mL against HCT-116 and MCF-7, respectively; which is 3.8 and 6.5-fold (based on the least IC_50_ values) more significant relative to the reference chemotherapeutic drug doxorubicin. In HepG-2 cells, the analogue 13h exhibited the highest cytotoxicity with IC_50_ value of 19.2µg/mL relative to doxorubicin (IC_50_ = 21.6µg/mL). The observed cytotoxicity was specific to cancerous cells, as evidenced by the minimal toxicity in the noncancerous control skin-fibroblast cells. ELISA results indicated that the observed antiproliferative effect against examined cancer cell lines is mediated *via* engaging the activation of apoptosis as illustrated by the significant increase in proapoptotic protein markers (p53, bax and caspase-3) and reduction in the antiapoptotic marker bcl-2. Taken together, results of the present study emphasize the potential of spiro pyrazole-oxindole analogues as valuable candidate anticancer agents against human cancer cells.

## 1. Introduction

Cancer is a primary global burden disease that is classified as the second-leading cause of death after cardiovascular diseases [1,2]. The toxicity of the currently available repertoire of anticancer drugs and the inefficiency of chemotherapies are two major limitations in the battle against cancer. Therefore, designing and discovering effective and selective antitumor agents remains the primary objective in organic medical chemistry known as targeted therapeutic strategies [3]. One of these strategies is apoptosis a.k.a. programmed cell death that is considered an essential mechanism by the body to eliminate unwanted cells [4,5]. Therefore, triggering apoptosis in cancer cells will lead to immediate death and thereby increasing the control of cancer proliferation [4,5]. Methodically understanding the mechanism of apoptosis discloses that it is influenced by the expression of caspases, Bcl-2 family proteins, including either antiapoptotic or proapoptotic members [6,7]. Induction of apoptosis is considered as one of the most successful strategies to target cancer [4,5,6,7].

Isatin and its derivatives are a promising class of heterocyclic molecules that have diverse biological activities of interest [8]. The most common application of isatins in organic synthesis is primarily focused on the highly reactive C-3 carbonyl group [9]. The vast majority of reactions of the C-3 carbonyl group of isatins are nucleophilic additions or spiroannulation, which transform it into 2-oxindole derivatives [9]. To this end, 2-Oxindoles, particularly those that are spiro-fused to other cyclic frames, have attracted significant research interest in the disciplines of synthetic organic chemistry and medicinal chemistry [10,11,12,13,14]. They represent the core structures in a variety of natural products and drugs, such as isomitraphylline, isocorynoxine, isorhnchophlline, mitraphylline and uncarine [10,11,12,13,14]. Interestingly spirocyclic oxindoles or spiro-oxindoles combined to a rigid heterocyclic ring at C-3 of the oxindole core are the most efficacious class of small molecules. They inhibit cell proliferation, induce apoptosis in cancer cells and lead to tumor growth regression without affecting normal cells [15,16,17]. For example, it has been documented that oxindole alkaloids derived from the root bark of *Uncaria tomentosa* plants exhibit apoptosis-mediated cytotoxicity against acute lymphoblastic leukaemia cells [18].

In this context, the objectives of the current study are three-fold: *(**i**)* to design and synthesize a novel series of spiro pyrazole-3,3’-oxindole analogues; *(**ii**)* to evaluate their antimicrobial, antioxidant and antiproliferative bioactivity against three cancer cell line types (i.e., MCF-7, HCT-116 and HepG-2) and *(**iii**)* to determine the underlying mechanism to induce apoptosis in MCF-7 and HCT-116 cancer cells.

## 2. Results and Discussion

### 2.1. Chemistry

**Figure 1:**
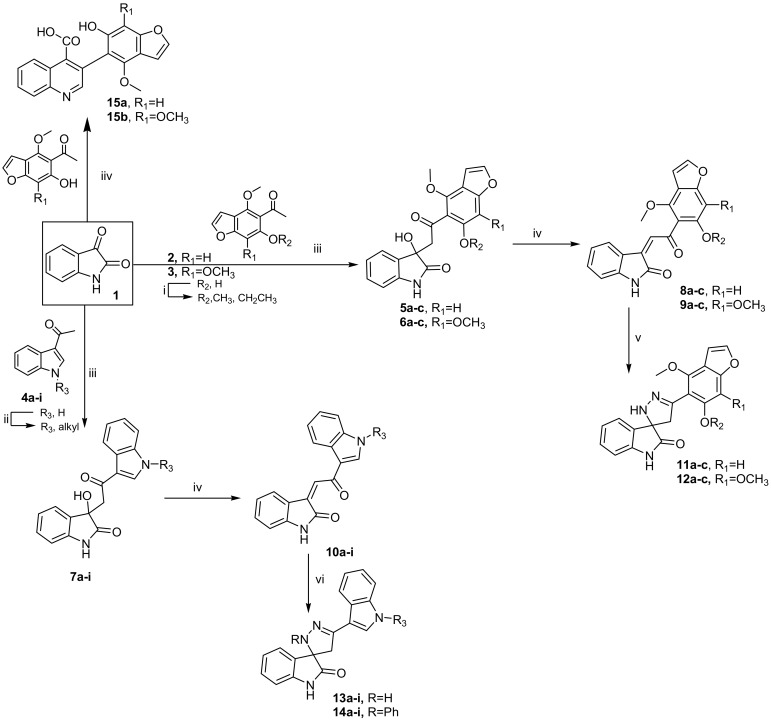
Reagents and conditions: (**i**) alkyl halide, acetone, K_2_CO_3_, reflux; (**ii**) alkyl halide, DMSO, NaOH, stirring, r.t.; (**iii**) EtOH, diethylamine, stirring, r.t. 10–15 days (Method A); EtOH, diethylamine, reflux, ~ 5h (Method B); (**iv**) gl. AcOH, HCl (2 drops), 80 °C, 30 min; (**v**) N_2_H_4_.H_2_O (98%), EtOH, gl. AcOH (2 drops); (**vi**) N_2_H_4_.H_2_O (98%) or NH_2_NHPh, EtOH, gl.AcOH (2 drops), reflux and (**vii**) EtOH, KOH (33%), reflux.

A simple and convenient route for the synthesis of 5’-(substituted)-2’,4’-dihydrospiro(indoline-3,3’-pyrazol)-2-ones and quinoline-4-carboxylic acids was summed up in Figure 1. Different aryl methyl ketones, namely 1-(6-substituted-4-methoxybenzofuran-5-yl)-ethan-1-ones 2a–c [19,20,21], 1-(6-substituted-4,7-dimethoxybenzofuran-5-yl)-ethan-1-ones 3a–c [19,20,21] and N-substituted-3-indolyl methyl ketones 4a–i [22,23], were prepared as starting materials for this study. Aldol condensation of isatin (1) with each aryl methyl ketones 2a–c, 3a–c and 4a–i in the presence of a catalytic amount of diethylamine under stirring for approximately 10–15 days at room temperature yielded the resultant aldol products: 3-hydroxy-3-(2-(aryl)-2-oxoethyl) indolin-2-ones 5a–c, 6a–c and 7a–i. 

Compounds 5a–c, 6a–c and 7a–i were also formed when the reaction was carried out in the presence of a few drops of diethylamine under reflux for ~ 5 h, which gave products identical in all aspects (mp, admixed mp) with no difference in their yields. Acid dehydration of the latter compounds using glacial acetic acid containing few drops of conc. HCl afforded the corresponding molecular hybrid 3-(2-(aryl)-2-oxo-ethylidene)indolin-2-ones 8a–c, 9a–c and 10a–i, respectively.

Cyclization of compounds 8a–c and 9a–c upon heating with hydrazine hydrate in dry ethanol and in the presence of a few drops of glacial acetic acid as a catalyst yielded the consequent 2’,4’-dihydrospiro(indoline-3,3’-pyrazol)-2-one derivatives 11a–c and 12a–c. Meanwhile, cyclization of 10a–i with hydrazine hydrate and/or phenyl hydrazine based on the above-provided method produced the corresponding 2’,4’-dihydrospiro(indoline-3,3’-pyrazol)-2-ones 13a–i and 2’-phenyl-2’,4’-dihydrospiro(indoline-3,3’-pyrazol)-2-ones 14a–i, respectively. 

On the other side, the reaction of isatin (1) with 2a and/or 3a under action of aqueous potassium hydroxide 33% (Pfitzinger condition [24]) resulted in opening of the 2-oxopyrolidine ring of isatin and then re-cyclized to give 4-quinoline carboxylic acids 15a and 15b, correspondingly. The analytical and spectral data of the entire target compounds were compatible with their structures; see experimental part. 

### 2.2. Biological Activity

#### 2.2.1. Antimicrobial Activity

The newly synthesized compounds were chosen to be evaluated in vitro towards a variety of pathogenic microorganisms; Gram-positive bacteria: *S. aureus* (ATCC 6538) and *B. subtilis* (ATCC 6633); Gram-negative bacteria: *P. areuginosa* (ATCC 27853) and *E. coli* (DSMZ 1058); yeast: *C. albicans* (ATCC 10231) and *S. cerevisiae* (ATCC 9080) and fungi: *A. niger* (NRRL A-326) using the disk diffusion method at a single dose of 20 µl. The results are shown in Table 1 as the growth inhibition zone (mm). Compounds 13a and 13b exhibited potent activity against *C. albicans* and *S. cerevisiae* with an inhibition zone of 18, 18, 20 and 18 mm compared to the reference drug amphotericin B of 24.8 and 23.5 mm. Besides, 11a, 11b and 11c showed significant inhibition zones of 20, 18 and 20 mm, respectively, towards *C. albicans*. Meanwhile, 11c has the most potent activity, with a growth inhibition zone of 25 mm higher than the reference drug amphotericin B of 23.5 mm towards *C. albicans*. On the other hand, compounds 12a, 12b, 12c and 15a resulted in significant growth inhibition zones of 16, 18, 18 and 16 mm, respectively, against B. subtilis compared to the reference drug amoxicillin of the growth inhibition zone 28.4 mm. While 12b, 12c and 15a revealed significant activity against *P. areuginosa* with growth inhibition zones of 24, 20 and 24 mm, respectively, compared to ciprofloxacin with a zone inhibition value of 30.2 mm (Table 1). The rest of the tested compounds exhibited trivial or no effects on the pathogenic microorganisms under investigation.

#### 2.2.2. Antioxidant Activity

Antioxidant activity of the newly synthesized 13a–i, 14a–i, 11a–c and 12a–c were assessed in terms of hydrogen-donating or radical-scavenging ability via the stable free radical 1,1-diphenyl-2-picrylhydrazyl (DPPH) using ascorbic acid as a reference at a concentration of 20µg/l. The results indicate that the ability of the tested compounds to reduce a solution of violet DPPH radical, rendering it colorless, increases after 15 to 60 min. Compounds 13a, 13b, 13g, 13i, 14a, 14b, 14d and 14i exhibited moderate free radical-scavenging effects after 60 min, ranging from 53.67% to 69.18% compared to ascorbic acid of 99.67%. Notably, compounds 12h and 12a revealed potent free radical-scavenging activity after 60 min that is equivalent to of 85.99% and 81.41%, respectively, compared to 99.67% given by a standard solution of ascorbic acid (Table 2).

#### 2.2.3. Evaluation of In Vitro Anti-Proliferative Activity 

The newly synthesized compounds 11a–c, 12a–c, 13a–i and 14a–i were screened for their antiproliferative activity against human breast cancer (MCF-7), human colon cancer (HCT-116) and human liver cancer (HepG2), as well as the normal skin fibroblast cell (BJ-1) through *in vitro* MTT assay (Table 3). The obtained results indicated that all congeners under investigation exhibited selective cytotoxicity against HCT-116 and MCF-7 cell lines as compared to the reference doxorubicin, with antiproliferative activity ranging from 84.6% to 97.9%. Minimal cytotoxicity was observed against normal skin fibroblast cells (BJ-1). With reference to MCF-7 cells, all compounds showed potent antiproliferative activity ranging from 79.8% to 97.4%, as compared to the positive control drug doxorubicin, except compounds **14i**, **11c** and **12c**, which exhibited low antiproliferative activity within the range of 52.3%–0%. Regarding the HepG-2 cancer cell line, only compounds 13a–i have been investigated, showing potent antiproliferative activity ranging from 72.8% to 96.8%. Interestingly, most of the tested analogues revealed minimal inhibition effects ranging from 16.6% to 5.0% against normal skin fibroblast cells (BJ-1), with the exception of compound 13i (69.4%). This finding encouraged the belief that these compounds possess high selectivity against cancer cells and high safety margins against normal cells, thereby highlighting their utilities as potent and safe anticancer drugs for further studies.

**Figure 2:**
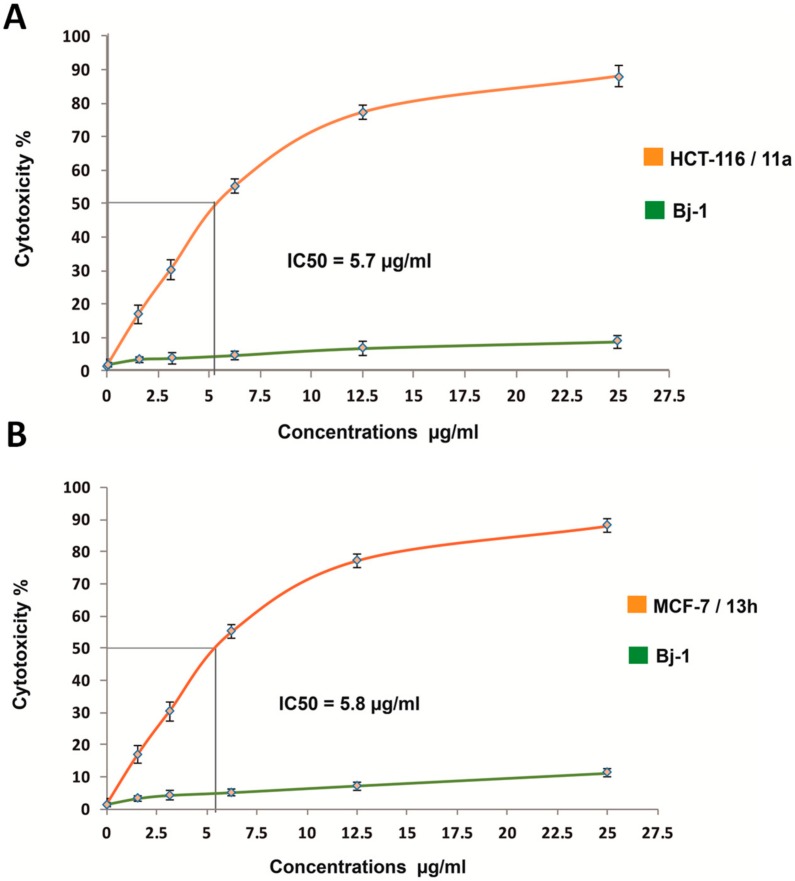
Cytotoxicity (%) dose-response curves of increasing concentrations for selected synthesized spiro pyrazole-oxindole congeners against cancerous cells. (**A**) Dose-response curve of **11a** congener against HCT-116 as compared to BJ-1. (**B**) (**A**) Dose-response curve of 13h congener against MCF-7 as compared to BJ-1. IC50 values in µg/mL were calculated by probit analysis using an Excel-based macro.

The dose-response cytotoxicity curves for selected drugs with potent antiproliferative activity (i.e., 11a and 13h) with reference to the BJ-1 line, are depicted in Figure 2.

The concentration required for 50% inhibition of cell viability (IC_50_) has been calculated for the most active compounds that exhibited low toxicity against BJ-1 cells using doxorubicin as a reference drug (Table 4). Interestingly, the IC_50_ values of compounds 11a, 11b, 12a, 12b, 13b, 13c and 13h were shown to be evidently more potent than the reference doxorubicin of IC_50_ = 21.6µg/mL against the HCT-116 cancer cell line. Their activity were in the descending order of 11a > 12a > 12b > 13c > 11b > 13h > 13b with IC_50_ of 5.7, 5.8, 7.9, 14.7, 16.4, 20.5 and 21.3µg/mL, respectively. Moreover, data revealed that compound 13h induced the highest cytotoxicity against the HepG-2 cancer cell line, with IC_50_ of 19.2µg/mL as compared to the reference doxorubicin with IC_50_ of 21.6µg/mL. On the other hand, most of the tested compounds were found to exhibit potent cytotoxicity with IC_50_, values ranging from 5.8 to 37.4ug/mL towards the MCF-7 cancer cell line relative to the reference doxorubicin with IC_50_ of 37.6µg/mL. The most potent drugs were 13h and 12b, with exhibited IC_50_ values of 5.8 and 16.7µg/mL, respectively.

#### 2.2.4. Apoptosis Induction in MCF-7and HCT-116 Cancer Cells

The promising antiproliferative results reported for compounds 11a, 11b, 12a, 12b and 13c against cell lines under study have prompted us to investigate the mechanism of cell death induced by these compounds. Apoptosis induction was examined in both MCF-7 and HCT-116 cancer cells by assaying the level of its key protein markers, namely: proapoptotic (p53, Bcl 2-associated X, Bax and caspases3) and antiapoptotic (B-cell lymphoma-2, Bcl-2). MCF-7 and HCT-116 cells undergoing apoptosis was detected by use of the ELISA technique. Protein marker levels were quantified according to Bradford assay. The results indicated that compounds 11a, 11b, 12a and 12b significantly reduced the expression levels of the antiapoptotic protein Bcl-2 by ~ 50%, 63%, 52% and 51%, respectively, towards MCF-7 cells compared to the control (Table 5). Meanwhile, treatment of the HCT-116 cells with compounds 11a, 11b, 12a and 12b conspicuously reduced the expression levels of the antiapoptotic protein Bcl-2 by ~ 66%, 76%, 26%, and 29%, respectively, compared to the control (Table 6).

#### 2.2.5. Effect of the Newly Synthesized Compounds on Key Pro- and Antiapoptotic Markers

Cell apoptosis is a sophisticated meticulously regulated biological process that is associated with complex signaling pathway responses [7,25]. Caspases are a group of cysteine-aspartic acid proteases that play a pivotal role as in the execution and intracellular regulation of apoptosis [26]. Caspase-3 is a key executioner of apoptosis that is activated both extrinsically (death ligands) and intrinsically (mitochondrial dysfunction) [27,28]. On the other hand, the tumor suppressor protein p53 is a positive regulator of the Bcl-2-associated X (Bax), Bcl-2-associated death promoter (Bad) and Bcl-2 homologous antagonist/killer (Bak) proapoptotic proteins to prevent Bcl-2 capture [7,29]. Free Bax, Bad and Bak subsequently bind to the mitochondrial membrane to induce mitochondrial damage and cell apoptosis [30]. Zhang and his coworker have demonstrated that p53 promotes the transcription of Bax and Bak, which regulate the release of cytochrome c from the mitochondria and result in cell apoptosis by activating the cleaving of caspase-3 [7]. Therefore, the increase in the expression of P53 and Bax causes the increase of the protein expression levels of active caspase-3. In the present study, treatment of the MCF-7 cell with compounds 11a, 11b, 12a, 12b and 13c resulted in a significant elevation of active caspase-3 protein levels by ~ 1.6, 1.6, 1.4, 4.6 and 3.8 folds, respectively, compared to the control. In line with the previous, compounds 11a, 11b, 12a, 12b and 13c increased the expression levels of proapoptotic p53 and Bax compared to the control. In addition, treatment of the HCT-116 cell with compounds 11a, 11b, 12a, 12b and 13c gave rise of caspase-3 protein levels by ~ 1.5, 1.0, 1.3 and 2.4 folds, respectively, compared to the control, as well as increase the expressions of proapoptotic proteins p53 and Bax compared to the control. Despite that the compound 13c increased the expression of active caspase-3, p53 and Bax proteins, it caused an increase of the Bcl-2 protein level. This result suggests that, additional yet undefined regulatory mechanism might be present to fine-tune apoptosis induction by 13c treatment (Table 5), which merits further investigation.

In this context, an evident correlation between the magnitude of elevation in the Bax/Bcl-2 ratio that is proportional to that of caspase-3 upregulation was found. This correlation is regarded as a key signal that triggers the execution of apoptosis in pyrazole-3,3’-oxindole-treated cancer cells, thereby leading to the observed potent antiproliferative activity in an MTT assay.

In conclusion, the present work highlights the potential application of a novel series of spiro pyrazole-3,3’-oxindoles as powerful synthetic antiproliferative agents acting via the induction of apoptosis in MCF7 and HCT-116 cancer cell lines. Further thorough investigation is warranted in order to fully explore the spectrum of cytotoxicity of these novel spiro pyrazole-3,3’-oxindole analogues against a battery of cancerous cells lines, as well as their in vivo biological activity in experimental animal models.

## 3. Materials and Methods

### 3.1. Chemicals and Supplies

Unless otherwise stated, all chemicals, including MTT (3-[4,5-dimethylthiazol-2yl]-2.5-diphenylterazolium bromide) were purchased from Sigma Aldrich (St Louis, MO, USA). RPM1 1640, FBS, l-glutamine and penicillin/streptomycin were obtained from Hyclone Laboratories (Logan, UT, USA).

### 3.2. Chemical and Physical Characterization of Synthesized Spiro Pyrazole-Oxindole Congeners

All reagents and solvents were of analytical grade. Melting points were determined on the digital melting point apparatus (Electro thermal 9100, Electro thermal Engineering Ltd. serial No. 8694, Rochford, United Kingdom) and are uncorrected. The reaction progress was monitored by thin-layer chromatography (TLC) using silica gel plates (POLYGRAM SILG/UV254, 0.20 mm), which were visualized under UV light 254 and 365 nm. Elemental analyses were carried out on a Perkin-Elmer 2400 analyzer (Perkin-Elmer, PerkinElmer, Inc**,** Waltham, MA, United States) and were found within ± 0.4% of the theoretical values. IR spectra were implemented on a Beckman infrared spectrophotometer PU 7712 using a *KBr* disk. ^1^H and ^13^C NMR spectra were measured with a Bruker Advance spectrometer (Bruker, Germany) at 400 and 100 MHz, respectively, using TMS as the internal standard. Chemical shifts were represented as parts per million (ppm) relative to the solvent peak. Mass spectra (EI) were recorded on Jeol JMS-AX 500 (EI) 70ev (JEOL Ltd. 1-2, Musashino 3-chome Akishima, Tokyo 196-8558, Japan). 

### 3.3. Synthesis 

#### 3.3.1. General Procedure for the Preparation of 3-hydroxy-3-(2-(aryl)-2-oxoethyl)indolin-2-ones **5a**–**c**, **6a**–**c** and **7a**–**i**

Method A: To a solution of isatin (**1**) (10 mmol) and aryl methyl ketone **2a**–**c, 3a**–**c** and **4a**–**i** (10 mmol) in absolute ethanol (20 mL), diethylamine (10 mmol) was added. The reaction mixture was stirred at room temperature for approximately 10–15 days. The solid formed was filtered off, washed with water, air-dried and used in the next step without further purification. In the case of compounds **5a, 6a** and **7a**, the product was obtained only after stirring for 5h at room temperature and left overnight. 

Method B: A solution of isatin (**1**) (10 mmol) and aryl methyl ketone **2a**–**c, 3a**–**c** and **4a**–**i** (10 mmol) in absolute ethanol (20 mL) containing diethylamine (10 mmol) was heated under reflux for ~5 h. The progress of the reaction was monitored using TLC. After the reaction accomplished, the reaction mixture was cooled, and the solid that formed was filtered off, washed with water, air-dried and used in the next step without further purification. 

#### 3.3.2. General Procedure for the Preparation of 3-(2-(aryl)-2-oxo-ethylidene)indolin-2-ones **8a**–**c**, **9a**–**c** and **10a**–**i**

A solution of compound **5a**–**c, 6a**–**c** and **7a**–**i** in a mixture of glacial acetic acid (5 mL) and two drops of concentrated HCl was heated at 80 °C for 30 min. After cooling, the reaction mixture was quenched in ice-water. The solid that formed was filtered off, washed with water, air-dried and crystallized from ethyl acetate-cyclohexane.

*3-(2-(6-Hydroxy-4-methoxy-benzofuran-5-yl)-2-oxo-ethylidene)indolin-2-one* (**8a**)*:* Yield 54%; orange powder; mp 173–5 °C; IR (KBr, cm^−1^): 3421 (OH), 3176 (NH), 1705, 1685 (C=O), 1545 (C=C), 1132, 1043 (C-O-C); ^1^H NMR (300 MHz, DMSO-*d_6_*) δ: 11.77 (s, 1H, OH), 10.27 (s, 1H, NH), 7.82 (d, 1H, H-2 furan), 7.22 (d, 1H, Ar-H), 7.20 (m, 2H, Ar-H), 6.85-6.80 (m, 2H, Ar-H), 6.72 (s, 1H, CH), 6.63 (s, 1H, CH), 4.13 (s, 3H, OCH_3_); ^13^C NMR (75 MHz, DMSO-*d_6_*) δ: 192.71, 180.53, 161.19, 156.93, 155.94, 152.03, 146.82, 143.96, 130.04, 128.72, 127.16, 112.18,110.30, 105.91, 93.85, 62.65; EI-MS: *m/z* (%): 335 (M^+^, 15); Anal Calcd for C_19_H_13_NO_5_ (335.31): C, 68.06; H, 3.91; N, 4.18; found: C, 67.91; H, 3.61; N, 4.01.

*3-(2-(4,6-Dimethoxy-benzofuran-5-yl)-2-oxo-ethylidene)indolin-2-one* (**8b**): Yield 33%; reddish-brown powder; mp 97–2 °C; IR (KBr, cm^−1^): 3220 (NH), 1702, 1695 (C=O), 1565 (C=C), 1138, 1086 (C-O-C); ^1^H NMR (300 MHz, DMSO-*d_6_*) δ: 10.03 (s, 1H, NH), 7.92 (d, 1H, H-2 furan), 7.56 (d, 1H, Ar-H), 7.25-6.88 (m, 6H, Ar-H), 3.83 (s, 3H, OCH_3_), 3.77 (s, 3H, OCH_3_); Anal Calcd for C_20_H_15_NO_5_ (349.34): C, 68.76; H, 4.33; N, 4.01; found: C, 68.55; H, 4.21; N, 3.95.

*3-(2-(6-Ethoxy-4-methoxy-benzofuran-5-yl)-2-oxo-ethylidene)indolin-2-one* (**8c**): Yield 35%; reddish-brown powder; mp 88–90 °C; IR (KBr, cm^−1^): 3210 (NH), 1697, 1687 (C=O), 1555 (C=C), 1135, 1051 (C-O-C); ^1^H NMR (300 MHz, DMSO-*d_6_*) δ: 8.19 (s, 1H, NH), 7.97 (d, 1H, H-2 furan), 7.35–7.10 (m,6H, Ar-H), 6.99 (d, 1H), 5.19 (q, 2H, CH_2_), 4.03 (t, 3H, CH_3_), 3.77 (s, 3H, OCH_3_); ^13^C NMR (75 MHz, DMSO-*d_6_*) δ: 192.07, 180.23, 160.09, 156.71, 155.11, 150.93, 144.52, 140.06, 133.21, 129.11, 127.82, 112.06, 111.13, 150.62, 105.33, 92.57, 64.32, 62.00, 25.35; EI-MS: *m/z* (%): 363 (M^+^, 29); Anal Calcd for C_21_H_17_NO_5_ (363.11): C, 69.41; H, 4.72; N, 3.85; found: C, 69.14; H, 4.50; N, 3.62.

*3-(2-(6-Hydroxy-4,7-dimethoxy-benzofuran-5-yl)-2-oxo-ethylidene)indolin-2-one* (**9a**): Yield 35%; purple powder; mp 138–5 °C; IR (KBr, cm^−1^): 3421 (OH), 3201 (NH), 1707, 1695 (C=O), 1555 (C=C), 1119, 1053 (C-O-C); ^1^H NMR (300 MHz, DMSO-*d_6_*) δ: 12.35 (s, 1H, OH), 10.72 (s, 1H, NH), 8.42 (d, 1H, H-2 furan), 8.07 (d, 2H, Ar-H), 7.66–7.58 (m, 3H, Ar-H), 7.16 (s, 1H, CH), 3.96 (s, 3H, OCH_3_), 3.79 (s, 3H, OCH_3_); ^13^C NMR (75 MHz, DMSO-*d_6_*) δ: 190.82, 179.95, 160.33, 159.01, 155.63, 142.54, 128.21, 127.42, 122.51, 121.01, 111.70, 105.91, 61.55, 61.50; EI-MS: *m/z* (%): 365 (M^+^, 45); Anal Calcd for C_20_H_15_NO_6_ (365.09): C, 65.75; H, 4.14; N, 3.83; found: C, 65.38; H, 3.91; N, 3.51.

*3-(2-Oxo-2-(4,6,7-trimethoxy-benzofuran-5-yl)-ethylidene)indolin-2-one* (**9b**): Yield 60%; orange powder; mp 93–5 °C; IR (KBr, cm^−1^): 3155 (s, 1H, NH), 1705, 1685 (C=O), 1552 (C=C), 1160, 1095 (C-O-C); ^1^H NMR (300 MHz, DMSO-*d_6_*) δ: 10.79 (s, 1H, NH), 8.39 (d, 1H, H-2 furan), 8.05 (d, 1H, Ar-H), 7.39 (t, 1H, Ar-H), 7.25 (d, 1H, Ar-H), 7.14 (s, 1H, CH), 7.02 (t, 1H, Ar-H), 6.90 (d, 1H, Ar-H), 4.00, 3.98, 3.81 (3s, 9H, OCH_3_); ^13^C NMR (75 MHz, DMSO-*d_6_*) δ: 190.02, 180.12, 162.04, 156.77, 155.21, 149.63, 144.60, 130.91, 129.01, 127.81, 121.71, 121.04, 110.54, 105.92, 61.00, 60.25, 60.07; EI-MS: *m/z* (%): 379 (M^+^, 42); Anal Calcd for C_21_H_17_NO_6_ (379.11): C, 66.49; H, 4.52; N, 3.69; found: C, 66.15; H, 4.32; N, 3.44.

*3-(2-(6-Ethoxy-4,7-dimethoxy-benzofuran-5-yl)-2-oxo-ethylidene)indolin-2-one* (**9c**): Yield 33%; orange powder; mp 118–2 °C; IR (KBr, cm^−1^): 3212 (s, 1H, NH), 1698, 1686 (C=O), 1566 (C=C), 1154, 1033 (C-O-C); ^1^H NMR (300 MHz, DMSO-*d_6_*) δ: 10.23 (s, 1H, NH), 8.12 (d, 1H, H-2 furan), 7.85 (d, 1H, Ar-H), 7.35 (t, 1H, Ar-H), 7.27–6.97 (m, 4H, Ar-H), 5.12 (q, 2H, CH_2_), 4.03 (t, 3H, CH_3_), 3.98, 3.81 (2s, 6H, OCH_3_); Anal Calcd for C_22_H_19_NO_6_ (393.39): C, 67.17; H, 4.87; N, 3.56; found: C, 67.02; H, 4.71; N, 3.44.

*3-(2-(N-Ethyl-1H-indol-3-yl)-2-oxo-ethylidene)indolin-2-one* (**10b**):Yield 92%; yellow powder; mp 197–9 °C; IR (KBr, cm^−1^): 3100 (NH), 1695 (C=O), 1585 (C=C); ^1^H NMR (300 MHz, DMSO-*d_6_*) δ: 10.90 (s, 1H, NH indolin-2-one), 8.54 (s, 1H, H-2 indole), 8.38–8.35 (m, 2H, Ar-H), 7.95 (s, 1H, CH), 7.63–7.13 (m, 6H, Ar-H), 4.37 (t, 2H, CH_2_), 1.54 (q, 3H, CH_3_); EI-MS: *m/z* (%): 316 (M^+^, 32); Anal Calcd for C_20_H_16_N_2_O_2_ (316.35): C, 75.93; H, 5.10; N, 8.86; found: C, 75.73; H, 4.92; N, 8.65.

*3-(2-(N-Benzyl-1H-indol-3-yl)-2-oxo-ethylidene)indolin-2-one* (**10c**)*:* Yield 95 %, orange powder; mp 205-7 °C; IR (KBr, cm^-1^): 3162 (NH), 1692 (C=O), 1603 (C=C); ^1^H NMR (300 MHz, DMSO-*d_6_*) δ: 10.72 (s, 1H, NH), 8.87 (s, 1H, H-2 indole), 8.42–8.37 (m, 2H, Ar-H), 7.67 (s, 1H, CH), 7.54–6.98 (m, 11H, Ar-H), 5.65 (s, 2H, N-CH_2_); ^13^C NMR (75 MHz, DMSO-*d_6_*) δ: 189.37, 180.62, 159.35, 140.81, 138.46, 137,04, 130.98, 126.71, 123.45, 122.61, 122.12, 120.80, 120.68, 121.60, 121.32, 119.81, 42.03; EI-MS: *m/z* (%): 378 (M^+^, 42); Anal Calcd for C_25_H_18_N_2_O_2_ (378.42): C, 79.35; H, 4.79; N, 7.40; found: C, 79.14; H, 4.57; N, 7.22.

*3-(2-(N-Benzoyl-1H-indol-3-yl)-2-oxo-ethylidene)indolin-2-one* (**10d**):Yield 62%; brown powder; mp 252–4 °C; IR (KBr, cm^−1^): 3201 (NH), 1705, 1985 (C=O), 1576 (C=C); ^1^H NMR (300 MHz, DMSO-*d_6_*) δ: 10.72 (s, 1H, NH), 8.56 (s, 1H, H-2 indole), 8.42–8.35 (m, 2H, Ar-H), 7.68 (s, 1H, CH), 7.53–6.95 (m, 11H, Ar-H); EI-MS: *m/z* (%): 392 (M^+^, 12); Anal Calcd for C_25_H_16_N_2_O_3_ (392.41): C, 76.52; H, 4.11; N, 7.14; found: C, 76.35; H, 4.02; N, 6.93.

*3-(2-(N-(2-Chloro-benzoyl)-1H-indol-3-yl)-2-oxo-ethylidene)indolin-2-one* (**10e**): Yield 40%; light brown powder; mp 228–30 °C; IR (KBr, cm^−1^): 3167 (NH), 1707, 1665 (C=O), 1572 (C=C), 757 (C-Cl); ^1^H NMR (300 MHz, DMSO-*d_6_*) δ: 10.72 (s, 1H, NH indolin-2-one), 8.56 (s, 1H, H-2 indole), 8.38–8.33 (m, 2H, Ar-H), 7.67 (s, 1H, CH), 7.35–6.85 (m, 10H, Ar-H); EI-MS: *m/z* (%): 426/428 (M^+^/M+2, 5.87/3.1); Anal Calcd for C_25_H_15_ClN_2_O_3_ (426.85): C, 70.34; H, 3.54; N, 6.56; found: C, 70.01; H, 3.34; N, 6.23.

*3-(2-(N-(4-Chloro-benzoyl)-1H-indol-3-yl)-2-oxo-ethylidene)indolin-2-one* (**10f**):Yield 65%; brown powder; mp 269–71 °C; IR (KBr, cm^−1^): 3155 (NH), 1705, 1668 (C=O), 1555 (C=C), 752 (C-Cl); ^1^H NMR (300 MHz, DMSO-*d_6_*) δ: 10.72 (s, 1H, NH indolin-2-one), 8.42 (s, 1H, H-2 indole), 8.42–8.38 (m, 2H, Ar-H), 7.67 (s, 1H, CH), 7.53–6.95 (m, 10H, Ar-H); ^13^C NMR (75 MHz, DMSO-*d_6_*) δ: 192.50, 185.50, 178,92, 155.74, 152.03, 146.83, 143.91, 133.25, 130.04, 128.72, 127.78, 124.33, 122.98, 121.32, 112.18, 110.50; Anal Calcd for C_25_H_15_ClN_2_O_3_ (426.85): C, 70.34; H, 3.54; N, 6.56; found: C, 70.02; H, 3.21; N, 6.23.

*3-(2-(N-(4-Bromo-benzenesulfonyl)-1H-indol-3-yl)-2-oxo-ethylidene)indolin-2-one* (**10g**): Yield 50%; brown crystal; mp 267–9 °C; IR (KBr, cm^−1^): 3168 (NH), 1696 (C=O), 1581 (C=C), 1375, 1117 (SO_2_), 782 (C-Br); ^1^H NMR (300 MHz, DMSO-*d_6_*) δ: 10.72 (s, 1H, NH indolin-2-one), 8.56 (s, 1H, H-2 indole), 8.42–8.35 (m, 2H, Ar-H), 7.67 (s, 1H, CH), 7.53–6.86 (m, 10H, Ar-H); ^13^C NMR (75 MHz, DMSO-*d_6_*) δ: 188.56, 179.90, 152.74,145.72, 140.80, 138.53, 137.00, 135.42, 130.19, 127.19, 126.33, 123.92, 122.16, 120.59, 113.17, 110.40; Anal Calcd for C_24_H_15_BrN_2_O_4_S (507.36): C, 56.82; H, 2.98; N, 5.52; found: C, 56.61; H, 3.11; N, 5.73.

*3-(2-(N-(4-Chloro-benzenesulfonyl)-1H-indol-3-yl)-2-oxo-ethylidene)indolin-2-one* (**10h**): Yield 25%; brown powder; mp 276–8 °C; IR (KBr, cm^−1^): 3201 (NH), 1685 (C=O), 1576 (C=C), 1375, 1119 (SO_2_), 775 (C-Cl); ^1^H NMR (300 MHz, DMSO-*d_6_*) δ: 10.72 (s, 1H, NH indolin-2-one), 8.55 (s, 1H, H-2 indole), 8.39–8.35 (m, 2H, Ar-H), 7.67 (s, 1H, CH), 7.42–6.95 (m, 10H, Ar-H); EI-MS: *m/z* (%): 462/464 (M^+^/M+2, 33/11); Anal Calcd for C_24_H_15_ClN_2_O_4_S (462.90): C, 62.27; H, 3.27; N, 6.05; found: C, 62.01; H, 3.11; N, 5.86.

*3-(2-(N-(2-Nitro-benzenesulfonyl)-1H-indol-3-yl)-2-oxo-ethylidene)indolin-2-one* (**10i**): Yield 20%; reddish-brown powder; mp 189–90 °C; IR (KBr, cm^-1^): 3172 (NH), 1685 (C=O), 1555 (C=C), 1376, 1117 (SO_2_); ^1^H NMR (300 MHz, DMSO-*d_6_*) δ: 10.72 (s, 1H, NH indolin-2-one), 8.56 (s, 1H, H-2 indole), 8.45–8.35 (m, 2H, Ar-H), 7.67 (s, 1H, CH), 7.41–6.96 (m, 10H, Ar-H); EI-MS: *m/z* (%): 473 (M^+^, 5); Anal Calcd for C_24_H_15_N_3_O_6_S (473.46): C, 60.88; H, 3.19; N, 8.88; found: C, 60.52; H, 2.95; N, 8.53.

#### 3.3.3. General Procedure for the Synthesis of 2’,4’-dihydrospiro(indoline-3,3’-pyrazol)-2-one Derivatives **11a**–**c**, **12a**–**c** and **13a**–**i**

To a mixture of compound **8a**–**c, 9a**–**c** and **10a**–**i** (10 mmol) and hydrazine hydrate (50 mmol) in absolute ethanol (10 mL), a few drops of glacial acetic acid were added, and the reaction mixture was refluxed for 4–6 h. After cooling, the reaction mixture was poured onto ice-water, and the solid that formed was filtered off, washed with water, air-dried and crystallized from ethyl acetate-cyclohexane (1:1).

*5’-(6-Hydroxy-4-methoxy-benzofuran-5-yl)-2’,4’-dihydrospiro(indoline-3,3’-pyrazol)-2-one* (**11a**): Yield 87%; orange powder; mp 129–31 °C; IR (KBr, cm^−1^): 3554 (OH), 3228, 3155 (NH), 1699 (C=O), 1618 (C=N), 1550 (C=C), 1048, 1117 (C-O-C); ^1^H NMR (300 MHz, DMSO-*d_6_*) δ: 11.77 (s, 1H, OH), 10.99 (s, 1H, NH), 10.27 (s, 1H, NH), 7.93 (d, 1H, H-2 furan), 7.52 (d, 1H Ar-H), 7.34–7.02 (m, 3H, Ar-H), 6.97 (d, 1H, Ar-H), 6.74 (s, 1H, Ar-H), 5.95 (s, 2H, CH_2_), 4.14 (s, 3H, OCH_3_); ^13^C NMR (75 MHz, DMSO-*d_6_*) δ: 180.21, 165.35, 160.94, 154.62, 152.05, 145.36, 143.21, 132.32, 130.04, 128.57, 127.08, 122.06, 120.19, 105.91, 93.85, 64.50, 60.26, 45.56; EI-MS: *m/z* (%): 363 (M^+^, 13); Anal Calcd for C_19_H_15_N_3_O_4_ (349.34): C, 65.32; H, 4.33; N, 12.03; found: C, 65.17; H, 4.21; N, 11.92.

*5’-(4,6-Dimethoxy-benzofuran-5-yl)-2’,4’-dihydrospiro(indoline-3,3’-pyrazol)-2-one* (**11b**): Yield 65%; yellow powder; mp 180–2 °C; IR (KBr, cm^−1^): 3279, 3120 (NH), 1685 (C=O), 1617 (C=N) 1558 (C=C), 1060, 1139 (C-O-C); ^1^H NMR (300 MHz, DMSO-*d_6_*) δ: 11.02 (s, 1H, NH), 10.02 (s, 1H, NH), 8.01 (d, 1H, H-2 furan), 7.61 (d, 1H Ar-H), 7.51–6.66 (m, 5H, Ar-H), 5.52 (s, 2H, CH_2_), 4.14, 4.05 (2s, 6H, OCH_3_); EI-MS: *m/z* (%): 363 (M^+^, 12); Anal Calcd for C_20_H_17_N_3_O_4_ (363.37): C, 66.11; H, 4.72; N, 11.56; found: C, 66.01; H, 4.61; N, 11.44.

*5’-(6-Ethoxy-4-methoxy-benzofuran-5-yl)-2’,4’-dihydrospiro(indoline-3,3’-pyrazol)-2-one* (**11c**): Yield 51%; gray powder; mp 137–9 °C; IR (KBr, cm^−1^): 3222, 3124 (NH), 1693 (C=O), 1620 (C=N), 1562 (C=C), 1060, 1137 (C-O-C); ^1^H NMR (300 MHz, DMSO-*d_6_*) δ: 9.72 (s, 1H, NH), 8.29 (s, 1H, NH), 7.93 (d, 1H, H-2 furan), 7.62 (d, 1H Ar-H), 7.50 (t, 1H, Ar-H), 7.48 (d, 1H, Ar-H), 7.35–6.92 (m, 3H, Ar-H), 5.25 (s, 2H, CH_2_), 5.15 (q, 2H, OCH_2_), 4.03 (t, 3H, CH_3_), 3.77 (s, 3H, OCH_3_); Anal Calcd for C_21_H_19_N_3_O_4_ (377.39): C, 66.83; H, 5.07; N, 11.13; found: C, 66.65; H, 5.13; N, 11.02.

*5’-(6-Hydroxy-4,7-dimethoxy-benzofuran-5-yl)-2’,4’-dihydrospiro(indoline-3,3’-pyrazol)-2-one* (**12a**): Yield 45%; yellowish-brown powder; mp 270–2 °C; IR (KBr, cm^−1^): 3445 (OH), 3185, 3165 (NH), 1685 (C=O), 1618 (C=N), 1557 (C=C), 1153, 1132 (C-O-C); ^1^H NMR (300 MHz, DMSO-*d_6_*) δ: 10.67 (s, 1H, OH), 10.47, 10.23 (2s, 2H, 2NH), 7.95 (d, 1H, H-2 furan), 7.88 (d, 1H, Ar-H), 7.30–6.65 (m, 4H, Ar-H), 5.41 (s, 2H, CH_2_), 4.26, 4.19 (2s, 6H, OCH_3_); ^13^C NMR (75 MHz, DMSO-*d_6_*) δ: 184.52, 167.54, 160.19, 155.61, 145.52, 132.25, 130.05, 127.81, 127.06, 124.92, 121.25, 105.85, 63.92, 61.64, 60.25, 45.75; EI-MS: *m/z* (%): 379 (M^+^, 29); Anal Calcd for C_20_H_17_N_3_O_5_ (379.12): C, 63.32; H, 4.52; N, 11.08; found: C, 63.07; H, 4.33; N, 10.95.

*5’-(4,6,7-Trimethoxy-benzofuran-5-yl)-2’,4’-dihydrospiro(indoline-3,3’-pyrazol)-2-one* (**12b**): Yield 62%; orange powder; mp 145–7 °C; IR (KBr, cm^−1^): 3206, 3175 (NH), 1687 (C=O), 1618 (C=N), 1545 (C=C), 1163, 1034 (C-O-C); ^1^H NMR (300 MHz, DMSO-*d_6_*) δ: 12.31, 10.39 (s, 2H, 2NH), 8.40 (d, 1H, H-2 furan), 7.99 (d, 1H, Ar-H), 7.59 (t, 1H, Ar-H), 7.40–6.85 (m, 3H, Ar-H), 4.04 (s, 2H, CH_2_), 3.99, 3.92, 3.84 (3s, 9H, 3OCH_3_); Anal Calcd for C_21_H_19_N_3_O_5_ (393.39): C, 64.12; H, 4.87; N, 10.68; found: C, 64.01; H, 4.71; N, 10.53.

*5’-(6-Ethoxy-4,7-dimethoxy-benzofuran-5-yl)-2’,4’-dihydrospiro(indoline-3,3’-pyrazol)-2-one* (**12c**): Yield 42%; orange powder; mp 135–7 °C; IR (KBr, cm^−1^): 3210, 3165 (NH), 1685 (C=O), 1620 (C=N), 1555 (C=C), 1163, 1035 (C-O-C); ^1^H NMR (300 MHz, DMSO-*d_6_*) δ: 12.23, 10.20 (s, 2H, 2NH), 8.25 (d, 1H, H-2 furan), 7.78 (d, 1H, Ar-H), 7.41 (d, 1H, Ar-H), 7.25–7.13 (m, 2H, Ar-H), 6.85 (d, 1H, Ar-H), 5.50 (s, 2H, CH_2_), 5.02 (q, 2H, OCH_2_), 4.02 (t, 3H, CH_3_), 3.83, 3.76 (2s, 6H, OCH_3_); EI-MS: *m/z* (%):407 (M^+^, 22); Anal Calcd for C_22_H_21_N_3_O_5_ (407.42): C, 64.86; H, 5.20; N, 10.31; found: C, 64.71; H, 5.05; N, 10.17.

*5’-(1H-Indol-3-yl)-2’,4’-dihydrospiro(indoline-3,3’-pyrazol)-2-one* (**13a**): Yield 65%; orange powder; mp 318–20 °C; IR (KBr, cm^−1^): 3261, 3157 (NH), 1696 (C=O), 1618 (C=N), 1573 (C=C); ^1^H NMR (300 MHz, DMSO-*d_6_*) δ: 12.54, 11.87, 10.40 (3s, 3H, 3NH), 8.91 (d, 1H, Ar-H), 8.53 (s, 1H, H-2 indole), 8.39 (d, 1H, Ar-H), 8.17 (d, 1H, Ar-H), 7.57–7.12 (m, 5H, Ar-H), 5.45 (s, 2H, CH_2_); ^13^C NMR (75 MHz, DMSO-*d_6_*) δ: 179.75, 152.62, 142.66, 138.12, 127.81, 127.65, 124.62, 122.66, 121.32, 120.65, 112.05, 111.42, 62.50, 41.73; EI-MS: *m/z* (%): 302 (M^+^, 43); Anal Calcd for C_18_H_14_N_4_O (302.33): C, 71.51; H, 4.67; N, 18.53; found: C, 71.33; H, 4.31; N, 18.27.

*5’-(N-Ethyl-1H-indol-3-yl)-2’,4’-dihydrospiro(indoline-3,3’-pyrazol)-2-one* (**13b**): Yield 63%; orange powder; mp 143–5 °C; IR (KBr, cm^-1^): 3212, 3126 (NH), 1696 (C=O), 1618 (C=N), 1573 (C=C); ^1^H NMR (300 MHz, DMSO-*d_6_*) δ: 12.17, 10.91 (2s, 2H, 2NH), 8.93 (d, 1H, Ar-H), 8.54 (s, 1H, Ar-H), 8.38–7.13 (m, 7H, Ar-H), 4.37–4.19 (m, 4H, 2CH_2_), 1.54 (t, 3H, CH_3_); Anal Calcd for C_20_H_18_N_4_O (330.38): C, 72.71; H, 5.49; N, 16.96; found: C, 72.56; H, 5.32; N, 16.80.

*5’-(N-Benzyl-1H-indol-3-yl)-2’,4’-dihydrospiro(indoline-3,3’-pyrazol)-2-one* (**13c**): Yield 80%; orange powder; mp 83–5 °C; IR (KBr, cm^−1^): 3284, 3127 (NH), 1676 (C=O), 1618 (C=N), 1556 (C=C); ^1^H NMR (300 MHz, DMSO-*d_6_*) δ: 10.72, 8.92 (2s, 2H, 2NH), 8.53 (s, 1H, H-2 indole), 8.44 (m, 2H, Ar-H), 8.20 (t, 1H, Ar-H), 7.81 (d, 1H, Ar-H), 7.67 (d, 1H, Ar-H), 7.56–6.87 (m, 8H, Ar-H), 5.56, 5.50 (2s, 4H, 2CH_2_); ^13^C NMR (75 MHz, DMSO-*d_6_*) δ: 180.05, 151.75, 142.15, 138.55, 137.72, 130.01, 128.40, 127.21, 126.04, 122.72, 121.23, 120.07, 110.71, 63.00, 44.59, 41.35; EI-MS: *m/z* (%): 392 (M^+^, 43); Anal Calcd for C_25_H_20_N_4_O (392.45): C, 76.51; H, 5.14; N, 14.28; found: C, 76.51; H, 5.14; N, 14.28.

*5’-(N-Benzoyl-1H-indol-3-yl)-2’,4’-dihydrospiro(indoline-3,3’-pyrazol)-2-one* (**13d**): Yield 52%; brown powder; mp 307–9 °C; IR (KBr, cm^−1^): 3262, 3125 (NH), 1687 (C=O), 1618 (C=N), 1576 (C=C); ^1^H NMR (300 MHz, DMSO-*d_6_*) δ: 11.54, 10.72 (2s, 2H, 2NH), 8.55 (d, 1H, Ar-H), 8.39–8.33 (m, 2H, Ar-H), 7.53 (m, 2H, Ar-H), 7.34–6.86 (m, 9H, Ar-H), 5.05 (s, 2H, CH_2_); EI-MS: *m/z* (%): 406 (M^+^, 21); Anal Calcd for C_25_H_18_N_4_O_2_ (406.44): C, 73.88; H, 4.46; N, 13.78; found: C, 73.65; H, 4.32; N, 13.63.

*5’-(N-(2-Chloro-benzoyl)-1H-indol-3-yl)-2’,4’-dihydrospiro(indoline-3,3’-pyrazol)-2-one* (**13e**): Yield 45%; brown powder; mp 219–21 °C; IR (KBr, cm^-1^): 3212, 3156 (NH), 1707, 1687 (C=O), 1620 (C=N), 1535 (C=C), 752 (C-Cl); ^1^H NMR (300 MHz, DMSO-*d_6_*) δ: 11.55, 10.72 (2s, 2H, NH), 8.23 (s, 1H, H-2 indole), 8.02 (m, 2H, Ar-H), 7.85 (d, 1H, Ar-H), 7.35–6.85 (m, 9H, Ar-H), 4.67 (s, 2H, CH_2_); ^13^C NMR (75 MHz, DMSO-*d_6_*) δ: 190.00, 179.73, 151.82, 142.62, 140.16, 139.21, 138.50, 134.21, 129.44, 127.05, 122.91, 122.51, 120.16, 112.56, 110.71, 62.65, 42.08; Anal Calcd for C_25_H_17_ClN_4_O_2_ (440.88): C, 68.11; H, 3.89; N, 12.71; found: C, 68.02; H, 3.77; N, 12.63.

*5’-(N-(4-Chloro-benzoyl)-1H-indol-3-yl)-2’,4’-dihydrospiro(indoline-3,3’-pyrazol)-2-one* (**13f**): Yield 50%; off-white crystals; mp 202–4 °C; IR (KBr, cm^-1^): 3205, 3154 (NH), 1705, 1688 (C=O), 1618 (C=N), 1575 (C=C), 752 (C-Cl); ^1^H NMR (300 MHz, DMSO-*d_6_*) δ: 10.72, 9.52 (2s, 2H, 2NH), 8.32 (s, 1H, H-2 indole), 8.12 (m, 2H, Ar-H), 7.87 (m, 3H, Ar-H), 7.61–6.85 (m, 7H, Ar-H), 5.01 (s, 2H, CH_2_); EI-MS: *m/z* (%): 440/442 (M^+^, 21/7); Anal Calcd for C_25_H_17_ClN_4_O_2_ (440.88): C, 68.11; H, 3.89; N, 12.71; found: C, 67.94; H, 3.67; N, 12.56.

*5’-(N-(4-Bromo-benzenesulfonyl)-1H-indol-3-yl)-2’,4’-dihydrospiro(indoline-3,3’-pyrazol)-2-one* (**13g**):Yield 35%; orange powder; mp 190–2 °C; IR (KBr, cm^-1^): 3268 (NH), 1687 (C=O), 1620 (C=N), 1585 (C=C), 1367, 1132 (SO_2_), 785 (C-Br); ^1^H NMR (300 MHz, DMSO-*d_6_*) δ: 11.52, 10.72 (2s, 2H, 2NH), 8.31 (s, 1H, H-2 indole), 8.02–7.95 (m, 2H, Ar-H), 7.64–6.91 (m, 10H, Ar-H), 4.75 (s, 2H, CH_2_); ^13^C NMR (75 MHz, DMSO-*d_6_*) δ: 178.91, 152.05, 142.52, 140.01, 138.06, 137.27, 130.47, 129.42, 127.18, 122.06, 121.15, 120.11, 110.07, 64.00, 42.21; Anal Calcd for C_24_H_17_BrN_4_O_3_S (521.39): C, 55.29; H, 3.29; N, 10.75; found: C, 55.29; H, 3.29; N, 10.75.

*5’-(N-(4-Chloro-benzensulfonyl)-1H-indol-3-yl)-2’,4’-dihydrospiro(indoline-3,3’-pyrazol)-2-one* (**13h**): Yield 35%; orange powder; mp 197–9 °C; IR (KBr, cm^-1^): 3251, 3153 (NH), 1675 (C=O), 1618 (C=N), 1565 (C=C), 1345, 1132 (SO_2_), 775 (C-Cl); ^1^H NMR (300 MHz, DMSO-*d_6_*) δ: 9.75, 10.72 (2s, 2H, 2NH), 8.45 (s, 1H, H-2 indole), 7.99–6.85 (m, 12H, Ar-H), 5.61 (s, 2H, CH_2_); EI-MS: *m/z* (%): 476/478 (M^+^/M+2, 27/9); Anal Calcd for. C_24_H_17_ClN_4_O_3_S (476.93): C, 60.44; H, 3.59; N, 11.75; found: C, 60.28; H, 3.41; N, 11.57.

*5’-(N-(2-Nitro-benzensulfonyl)-1H-indol-3-yl)-2’,4’-dihydrospiro (indoline-3,3’-pyrazol)-2-one* (**13i**):Yield 35%; brown powder; mp 210–2 °C; IR (KBr, cm^-1^): 3227, 3156 (NH), 1685 (C=O), 1618 (C=N), 1575 (C=C), 1376, 1117 (SO_2_); ^1^H NMR (300 MHz, DMSO-*d_6_*) δ: 11.51, 10.72 (2s, 2H, 2NH), 8.50 (s, 1H, H-2 indole), 8.37–7.14 (m, 12H, Ar-H), 5.32 (2s, 2H, CH_2_); EI-MS: *m/z* (%): 487(M^+^, 21); Anal Calcd for. C_24_H_17_N_5_O_5_S (487.49): C, 59.13; H, 3.51; N, 14.37; found: C, 59.01; H, 3.43; N, 14.26.

#### 3.3.4. General Procedure for the Synthesis of 2’-phenyl-2’,4’-dihydrospiro(indoline-3,3’-pyrazol)-2-ones 14a–i

Compounds **14a**–**i** prepared according to previously described methods for compounds **13a**–**i** using an equimolar mixture of compounds **10a**–**i** (10 mmol) and phenyl hydrazine (50 mmol).

*5’-(1H-Indol-3-yl)-2’-phenyl-2’,4’-dihydrospiro(indoline-3,3’-pyrazol)-2-one* (**14a**): Yield 52%; orange powder; mp 124–6 °C; IR (KBr, cm^-1^): 3186 (NH), 1701 (C=O), 1620 (C=N), 1565 (C=C); ^1^H NMR (300 MHz, DMSO-*d_6_*) δ: 11.62, 10.72 (2s, 2H, 2NH), 8.22 (d, 1H, Ar-H), 8.05 (s, 1H, H-2), 7.92 (m, 1H, Ar-H), 7.89 (d, 1H, Ar-H), 7.82 (d, 1H, Ar-H), 7.64 (m, 1H, Ar-H),7.53 (d, 1H, Ar-H), 7.46–7.12 (m, 7H, Ar-H), 5.28 (s, 2H, CH_2_); ^13^C NMR (75 MHz, DMSO-*d_6_*) δ: 197.72, 152.31, 144.05, 142.15, 139.61, 137.72, 128.46, 128.04, 127.26, 122.71, 121.36, 121.01, 110.07, 68.71, 40.95; EI-MS: *m/z* (%):378 (M^+^, 22); Anal Calcd for. C_24_H_18_N_4_O (378.43): C, 76.17; H, 4.79; N, 14.81; found: C, 76.02; H, 4.61; N, 14.65. 

*5’-(N-Ethyl-1H-indol-3-yl)-2’-phenyl-2’,4’-dihydrospiro(indoline-3,3’-pyrazol)-2-one* (**14b**): Yield 51%; reddish-brown powder; mp 108–10 °C; IR (KBr, cm^-1^): 3196 (NH), 1678 (C=O), 1618 (C=N), 1556 (C=C); ^1^H NMR (300 MHz, DMSO-*d_6_*) δ: 10.52 (s, H, NH), 8.2 (d, 1H, Ar-H), 8.22 (s, 1H, Ar-H), 8.04–7.12 (m, 7H, Ar-H), 5.21 (s, 2H, CH_2_), 4.21 (q, 2H, CH_2_), 1.54 (t, 3H, CH_3_); EI-MS: *m/z* (%): 406 (M^+^, 22); Anal Calcd for C_26_H_22_N_4_O (406.48): C, 76.83; H, 5.46; N, 13.78; found: C, 76.71; H, 5.26; N, 13.50.

*5’-(N-Benzyl-1H-indol-3-yl)-2’-phenyl-2’,4’-dihydrospiro(indoline-3,3’-pyrazol)-2-one* (**14c**): Yield 60%; brown powder; mp 96–8 °C; IR (KBr, cm^-1^): 3165 (NH), 1676 (C=O), 1618 (C=N), 1558 (C=C); ^1^H NMR (300 MHz, DMSO-*d_6_*) δ: 10.72 (s, H, NH), 8.24 (s, 1H, H-2 indole), 8.12 (m, 2H, Ar-H), 7.98–7.02 (m, 16H, Ar-H), 5.55, 5.52 (2s, 4H, 2CH_2_); ^13^C NMR (75 MHz, DMSO-*d_6_*) δ: 179.91, 153.065–110.09, 69.11, 43.35, 41.04; EI-MS: *m/z* (%): 468 (M^+^, 12); Anal Calcd for C_31_H_24_N_4_O (468.55): C, 79.46; H, 5.16; N, 11.96; found: C, 79.46; H, 5.16; N, 11.96.

*5’-(N-Benzoyl-1H-indol-3-yl)-2’-phenyl-2’,4’-dihydrospiro(indoline-3,3’-pyrazol)-2-one* (**14d**): Yield 56%; brown powder; mp 100–2 °C; IR (KBr, cm^-1^): 3175 (NH), 1798, 1975 (C=O), 1534 (C=C); ^1^H NMR (300 MHz, DMSO-*d_6_*) δ: 10.45 (s, 1H, NH), 8.45 (s, 1H, H-2 indole), 8.06 (d, 1H, Ar-H), 7.98 (m, 2H, Ar-H), 7.85–7.02 (m, 15H, Ar-H), 5.50 (s, 2H, CH_2_); ^13^C NMR (75 MHz, DMSO-*d_6_*) δ: 187.60, 176.51, 151.91, 143.51, 142.24, 137.71, 136.15, 132.61, 130.01, 128.42, 127.91, 126.35, 122.36, 122.21, 121.31, 116.09, 112.14, 111.81, 68.95, 42.01; EI-MS: *m/z* (%): 482 (M^+^, 6); Anal Calcd for C_31_H_22_N_4_O_2_ (482.53): C, 77.16; H, 4.60; N, 11.61; found: C, 77.01; H, 4.52; N, 11.45.

*5’-(N-(2-Chlorobenzoyl)-1H-indol-3-yl)-2’-phenyl-2’,4’-dihydrospiro(indoline-3,3’-pyrazol)-2-one* (**14e**): Yield 40%; reddish-brown powder; mp 78–80 °C; IR (KBr, cm^-1^): 3176 (NH), 1710, 1695 (C=O), 1620 (C=N), 1555 (C=C), 755 (C-Cl); ^1^H NMR (300 MHz, DMSO-*d_6_*) δ: 10.72 (s, 1H, NH), 8.06 (m, 2H, Ar-H), 7.92 (d, 1H, Ar-H), 7.81–6.92 (m, 15H, Ar-H), 5.21 (s, 2H, CH_2_); EI-MS: *m/z* (%): 516/518 (M^+^/M^+^+2, 13/6); Anal Calcd for C_31_H_21_ClN_4_O_2_ (516.98): C, 72.02; H, 4.09; N, 10.84; found: C, 72.11; H, 4.19; N, 10.73.

*5’-(N-(4-Chlorobenzoyl)-1H-indol-3-yl)-2’-phenyl-2’,4’-dihydrospiro(indoline-3,3’-pyrazol)-2-one* (**14f**): Yield 52%; brown powder; mp 162–4 °C; IR (KBr, cm^-1^): 3210 (NH), 1797, 1685 (C=O), 1618 (C=N), 1575 (C=C), 757 (C-Cl); ^1^H NMR (300 MHz, DMSO-*d_6_*) δ: 10.72 (s, 1H, NH), 8.55 (1H, d, Ar-H), 8.21 (s, 1H, H-2 indole), 8.01 (m, 2H, Ar-H), 7.92–6.75 (m, 14H, Ar-H), 5.56 (s, 2H, CH_2_); EI-MS: *m/z* (%): 516/518 (M^+^/M^+^+2, 11/4); Anal Calcd for C_31_H_21_ClN_4_O_2_ (516.98): C, 72.02; H, 4.09; N, 10.84; found: C, 71.93; H, 3.98; N, 10.71.

*5’-(N-(4-Bromo-benzenesulfonyl)-1H-indol-3-yl)-2’-phenyl-2’,4’-dihydrospiro(indoline-3,3’-pyrazol)-2-one* (**14g**): Yield 61%; reddish-brown powder; mp 132–4 °C; IR (KBr, cm^-1^): 3186 (NH), 1692 (C=O), 1618 (C=N), 1555 (C=C), 1353, 1123 (SO_2_), 787 (C-Br); ^1^H NMR (300 MHz, DMSO-*d_6_*) δ: 10.54 (s, 1H, NH), 8.78 (d, 1H, Ar-H), 8.22 (s, 1H, H-2 indole), 8.14 (m, 2H, Ar-H), 7.88 (d, 1H, Ar-H), 7.77–6.87 (m, 13H, Ar-H), 5.25 (s, 2H, CH_2_); ^13^C NMR (75 MHz, DMSO-*d_6_*) δ: 180.05, 152.72, 144.35, 142.20, 139.51, 138.27, 136.06, 130.25, 128.26, 126.05, 122.31, 121.16, 112.53, 110.04, 112.55, 68.62, 41.16; Anal Calcd for C_30_H_21_BrN_4_O_3_S (597.48): C, 60.31; H, 3.54; N, 9.38; found: C, 60.24; H, 3.41; N, 9.22.

*5’-(N-(4-Chloro-benzenesulfonyl)-1H-indol-3-yl)-2’-phenyl-2’,4’-dihydrospiro(indoline-3,3’-pyrazol)-2-one* (**14h**): Yield 54%; orange powder; mp 110–2 °C; IR (KBr, cm^-1^): 3175 (NH), 1687 (C=O), 1618 (C=N), 1575 (C=C), 1355, 1132 (SO_2_), 775 (C-Cl); ^1^H NMR (300 MHz, DMSO-*d_6_*) δ: 10.72 (s, 1H, NH), 8.75 (d, 1H, Ar-H), 8.35 (s, 1H, H-2 indole), 8.11 (m, 2H, Ar-H), 7.98 (d, 1H, Ar-H), 7.87–6.75 (m, 13H, Ar-H), 5.54 (s, 2H, CH_2_); EI-MS: *m/z* (%): 553/555 (M^+^/M+2, 13/5); Anal Calcd for C_30_H_21_ClN_4_O_3_S (553.03): C, 65.15; H, 3.83; N, 10.13; found: C, 65.01; H, 3.72; N, 10.02.

*5’-(N-(2-Nitro-benzenesulfonyl)-1H-indol-3-yl)-2’-phenyl-2’,4’-dihydrospiro(indoline-3,3’-pyrazol)-2-one* (**14i**): Yield 41%; reddish-brown powder; mp 152–4 °C; IR (KBr, cm^-1^): 3177 (NH), 1695 (C=O), 1620 (C=N), 1585 (C=C), 1353, 1127 (SO_2_); ^1^H NMR (300 MHz, DMSO-*d_6_*) δ: 10.72 (s, 1H, NH), 8.57 (d, 1H, Ar-H), 8.15 (s, 1H, H-2 indole), 7.92 (m, 2H, Ar-H), 7.87 (d, 1H, Ar-H), 7.82 (m, 2H, Ar-H), 7.53–7.05 (m, 11H, Ar-H), 5.65 (2s, 2H, CH_2_); EI-MS: *m/z* (%): 563 (M^+^,24); Anal Calcd for C_30_H_21_N_5_O_5_S (563.58): C, 63.93; H, 3.76; N, 12.43; found: C, 63.75; H, 3.61; N, 12.35.

#### 3.3.5. General Procedure for Synthesis of Quinoline-4-carboxylic acids **15a**, **b**

To a solution of isatin (**1)** (10 mmol) in ethanol (10 mL), a solution of potassium hydroxide (10 mL, 33%) was added, and the mixture was heated under reflux for 10 min. After cooling, the reaction mixture was acidified to pH 2–3 using 0.38 mL of concentrated hydrochloric acid. To the reaction mixture, compound **2a** and **3a** (10 mmol) were added and then heated under reflux for 6–12 h. After cooling, the reaction mixture was poured into ice-water, and the solid formed was filtered off, washed with water, air-dried and crystallized from ethanol.

3-(6-Hydroxy-4-methoxybenzofuran-5-yl)quinoline-4-carboxylic acid (**15a**): Yield 70%; pale yellow powder; mp 217–9 °C; IR (KBr, cm^-1^): 3450 (OH), 1710 (C=O), 1595 (C=C), 1135, 1029 (C-O-C); ^1^H NMR (300 MHz, DMSO-*d_6_*) δ: 11.85 (s, 2H, 2OH), 8.47 (d, 1H, Ar-H), 8.30 (s, 1H, Ar-H), 8.13 (d, 1H, Ar-H), 7.82 (m, 3H, Ar-H), 7.17 (t, 1H, Ar-H), 6.91 (s, 1H, Ar-H), 4.51 (s, 3H, OCH_3_); ^13^C NMR (75 MHz, DMSO-*d_6_*) δ: 203.01, 167.53, 156.93, 155.62, 146.83, 143.52, 136.14, 128.61, 127.78, 125.38, 122.98, 122.09, 112.18, 110.33, 105.35, 93.35, 62.00; EI-MS: *m/z* (%):335 (M^+^, 33); Anal Calcd for C_19_H_13_NO_5_ (335.31): C, 68.06; H, 3.91; N, 4.18; found: C, 67.91; H, 3.84; N, 4.07.

*3-(6-Hydroxy-4,6-dimethoxybenzofuran-5-yl)quinoline-4-carboxylic acid* (**15b**): Yield 53%; yellow powder; mp 275–7 °C; IR (KBr, cm^-1^): 3510 (OH), 1709 (C=O), 1587 (C=C), 1155, 1031 (C-O-C); ^1^H NMR (300 MHz, DMSO-*d_6_*) δ: 12.31 (s, 2H, 2OH), 8.42 (d, 1H, Ar-H), 8.07 (d, 1H, Ar-H), 7.91 (s, 1H, Ar-H), 7.66–7.58 (m, 2H, Ar-H), 7.16 (s, 2H, Ar-H), 3.96, 3.79 (2s, 6H, 2OCH_3_); ^13^C NMR (75 MHz, DMSO-*d_6_*) δ: 200.93, 161.33, 152.70, 147.23, 143.61, 138.51, 132.71, 128.35, 126.90, 124.44, 123.66, 105.91, 61.50, 59.25; EI-MS: *m/z* (%):365 (M^+^, 33); Anal Calcd for C_20_H_15_NO_6_ (365.34): C, 65.75; H, 4.14; N, 3.83; found: C, 65.61; H, 4.03; N, 3.65.

### 3.4. Biological Assays

#### 3.4.1. Antimicrobial Evaluation

The antimicrobial activity of target compounds were evaluated to be in vitro against a variety of pathogenic microorganisms, namely *Staphylococcus aureus* (ATCC 6538)*, Bacillus subtilis* (ATCC 6633) (Gram-positive bacteria), *Pseudomonas areuginosa* (ATCC 27853), *Escherichia coli* (DSMZ 1058) (Gram-negative bacteria), *Candida albicans* (ATCC 10231), *Saccharomyces cerevisiae* (ATCC 9080) (Yeast) and *Aspergillus niger* (NRRL A-326) (Fungi) using the disk diffusion method [31] at a single dose of 20 µg. The microorganisms were served and obtained from the Microbial Biotechnology Department, National Research Center, Giza, Egypt. Amphotericin, amoxicillin and ciprofloxacin were used as reference drugs (Oxoid™ Disks). The tested compounds 11a–c, 12a–c, 13a–i, 14a–i and 15a,b were dissolved in dimethylsulphoxide (DMSO) at a concentration of 1 mg/mL. Aliquots of 20 μl were soaked on filter paper discs (6 mm) and dried at room temperature under sterilized conditions. The paper discs were placed on inoculated agar plats and then incubated for 24 h at 37 °C for bacterial strains and after 48–72 h incubation at 30 °C for fungi. Both bacterial and yeast strains were grown on nutrient agar medium (g/l): beef extract, 3; peptone, 10 and agar, 20 at pH of 7.2. The fungal strain was grown on Czapek-Dox medium (g/l): sucrose, 30; NaNO_3_, 3; MgSO_4_.7H_2_O, 0.5l; KCl, 0.5; FeSO_4_, 0.01; K_2_HPO_4_, 1 and agar, 20 at pH of 6.0. 

#### 3.4.2. Antioxidant Evaluation

The free radical scavenging activity (RSA) was assessed spectrophotometically at 517 nm via decoloration of the solution of the DPPH (1,1-diphenyl-2-picrylhydrazyl) radical according to Brand-Williams et al. 1995 [32]. DPPH solution was prepared by dissolving of 20 mg of DPPH in one liter of dimethylsulphoxide (DMSO). A freshly prepared DPPH solution was used for the assay. Each of the tested compounds 11a–c, 12a–c, 13a–i, 14a–i and 15a,b were dissolved in DMSO (1mg/mL). A volume of 20µg/l of a DMSO stock solution of the test compounds was added to 2 mL of freshly prepared DPPH in DMSO solution. The mixtures were shaken in a vortex (2500 rpm) for 1 min and then left to stand for 15, 30, 45 and 60 min in the dark room. The absorbance was measured at 517 nm against blank using UV visible spectrophotometer 2401 PC (Shimadzu, Kyoto, Japan). Lower absorbance of the reaction mixture indicated higher free radical scavenging activity was analyzed from the graph plots of the inhibition percentage against the compound concentration. The experiment was carried out in triplicate and averaged. The scavenging activity was calculated according to the following formula:

Scavenging ability (%) = (A_517 of control_ - A_517of sample /_ A _517 of control_) ×100 (1)

#### 3.4.3. Anticancer Evaluation

##### Cell Culture

MCF-7 (human breast cancer), HCT-116 (human colon cancer) and HepG2 (human liver cancer) cell lines were obtained from Karolinska Institute, Stockholm, Sweden. All cells were maintained in RPMI 1640 medium. The media were supplemented with 10% heat-inactivated fetal bovine serum plus 1% antibiotic-antimycoticmixture (10,000 U/mL of potassium penicillin, 10,000 μg/mL of streptomycin sulfate, 25 μg/mL of amphotericin B and 1% of *L* glutamine (Biowest, USA)). BJ-1 of a human skin fibroblast derived from normal foreskin was obtained from ATCC^®^ CRL-2522^™^ as a frozen ampoule with about 1 × 10^6^ cells per 1 ml volume. BJ-1 was maintained in MEM with 2 mM of *L*-glutamine and Earle’s salts medium. 

##### MTT Cytotoxicity Assay

Cell viability was studied using the MTT 3-(4,5-dimethylthiazol-2-yl)-2,5-diphenyl-tetrazolium bromide (Bio Basic Canada Inc. Toronto, Canada) assay [33]. The steps were executed in a sterile laminar air flow cabinet biosafety class II level (Baker, SG403INT; Sanford, ME, USA). All incubations were carried out at 37 °C in a 5% CO_2_ incubator under a humidified atmosphere of 95% (Sheldon, TC2323; Cornelius, OR, USA). Cells were seeded into 96-well microtiter plastic plates at a concentration of (10^4^ cells/well) and allowed to adhere for 24 h. The medium was aspirated and then added to the cells with the test compounds 11a–c, 12a–c, 13a–i, 14a–i and 15a,b at a single dose of 100 μg/mL in DMSO. After incubation of the medium for 48 h, 40 μL of MTT salt (2.5 μg/mL) was added to each well and then incubated for an additional 4 h. To stop the reaction and dissolve any formed formazan crystals, 200 μL of 10% sodium dodecyl sulfate (SDS) were added to each well and incubated overnight at 37 °C. The amount of formazan product was measured at 595 nm with a reference wavelength of 690 nm as a background using a microplate reader (Bio-Rad Laboratories, model 3350, California, USA). For the untreated cells (negative control), the medium was added instead of the tested compounds. A positive control adrinamycin^®^ (doxorubicin, Mr = 579.9) (Pharmacia India Pvt Ltd. Gurgaon, Haryana 122001, India) was used as a known cytotoxic natural agent giving 100% inhibition. Dimethylsulfoxide (DMSO) was the vehicle used for dissolution of the testing compound, and its final concentration on the cells was less than 0.2%. At the same dilution, the solvent concentration was the same for all drugs and between the control and drug treatments. The concentration required for 50% inhibition of cell viability (IC_50_) was calculated for the potent compounds that showed preliminary cytotoxic effects at 100μg/mL by applying various concentrations of 0, 10, 15, 20, 25, 30, 40 and 50μg/mL (three replicates per concentration group) of the tested compounds and employing the probit analysis method using a simple *t*-test (SPSS statistical analysis software package/version 11.0, SPSS Inc. (IL), Chicago, USA).

### 3.5. Apoptosis Assay

The cellular apoptosis protein markers were analyzed after treatment with the IC_50_ of 11a, 11b, 12a, 12b and 13c, which were characterized for their relevant antiproliferative activity previously. Briefly, cells (HepG2 and HCT) were seeded at a concentration of 1.2–1.8 × 10^3^ cells/well in 6-well plates. After 48-h treatment, the collected cells were lysed and centrifuged at 10,000 rpm for 20 min at 4 °C. The protein concentration was measured in the supernatant by Bradford protein assay [34]. Volume containing 50 mg of total protein was incubated with 5 mL of caspase substrate in 100 mL of the reaction buffer at 37 °C for 1 h in the dark. Caspase-3 activity was determined by a microplate reader at 405 nm using a caspase-3 colorimetric assay kit (Abcam) according to the manufacturer’s instructions [23]. *In vitro* protein level quantitative measurement of apoptotic marker p53, Bcl-2-associated X (Bax) and antiapoptotic marker B-cell lymphoma-2 (Bcl-2) in cell lysate were assessed by Enzyme-Linked Immunosorbent Assay Simple Step ELISA^®^ (ab199080, ab119506 and ab171571; Abcam) according to the manufacturer’s instructions [35,36,37].

Data are presented as means ± SD. Individual groups were compared using the two-tailed independent Student’s *t*-test. Multiple group comparisons were carried out using one-way analysis of variance (ANOVA) followed by the Tukey–Kramer test for post-hoc analysis.

## Figures and Tables

**Table 1:** Antimicrobial activity of the most active compounds (20 mg per disc).^a.^

Compd. No ^b^	Inhibition Zone (mm)						
	Gram-Positive		Gram-Negative		Yeast		Fungi
	*S. aureus* (ATCC 6538)	*B. subtilis* (ATCC 6633)	*P. aeruginosa* (ATCC 27853)	*E. coli* (DSMZ 1058)	*C. albicans* (ATCC 10231)	*S. cerevisiae* (ATCC 9080)	*A. niger* (NRRL A-326)
**11a**	-	12	8	-	20	16	-
**11b**	-	12	12	-	18	18	-
**11c**	-	10	10	-	20	25	-
**12a**	8	16	18	-	12	10	-
**12b**	10	18	24	-	10	10	8
**12c**	10	18	20	-	10	10	-
**13a**	-	-	-	-	18	20	-
**15a**	8	16	24	-	12	-	10
**15b**	8	10	-	-	8	-	-
**Amoxicillin**	25.6	28.4	-	-	-	-	-
**Ciprofloxacin**	-	-	30.2	25.8	-	-	-
**Amphotericin B**	-	-	-	-	24.8	23.5	26.7

^a^ disk diffusion method and ^b^ concentration 20 µg.

**Table 2:** Scavenging activity % on DPPH radicals of the most active compounds at a concentration of 20µg/l.

Compd. No	Scavenging Activity (%) ^a^ at Different Time (min)			
	15	30	45	60
**11a**	6.86 ± 1.17	10.26 ± 1.37	17.73 ± 1.28	26.87 ± 1.56
**11b**	9.60 ± 1.77	14.28 ± 1.52	20.44 ± 1.65	30.76 ± 2.04
**11c**	40.65 ± 1.28	40.65 ± 1.81	40.65 ± 1.67	40.65 ± 1.35
**12a**	56.43 ± 1.08	67.09 ± 1.45	76.92 ± 1.51	81.41 ± 1.37
**12b**	17.29 ± 1.53	23.46 ± 1.64	30.56 ± 1.49	40.65 ± 1.46
**12c**	9.76 ± 1.76	10.24 ± 1.26	11.39 ± 1.53	13.97 ± 1.55
**13a**	41.14 ±1.25	45.77 ± 1.36	56.55 ± 1.99	64.08 ± 2.01
**13b**	43.52± 1.98	47.05 ± 1.81	56.33 ± 1.29	65.55 ± 1.55
**13c**	13.97± 1.36	20.97 ± 1.26	23.96 ± 1.51	35.13 ± 1.61
**13d**	19.32± 1.24	31.32 ± 1.81	25.92 ± 1.24	39.70 ± 1.99
**13e**	9.10 ± 1.38	26.15 ± 1.26	36.70 ± 1.62	48.13 ± 1.82
**13f**	17.90 ± 1.62	28.28 ± 1.61	34.28 ± 1.27	40.74 ± 1.54
**13g**	25.95 ± 1.85	44.00 ± 1.46	49.25 ± 1.77	53.67 ± 1.81
**13h**	15.22 ± 1.98	16.31 ± 1.96	20.58 ± 1.48	35.71 ± 1.91
**14i**	45.88 ± 1.05	54.32 ± 1.08	60.60 ± 1.64	69.18 ± 1.65
**14a**	41.09 ± 2.05	51.52 ± 1.91	53.98 ± 2.06	59.22 ± 2.14
**14b**	42.36 ± 1.45	58.08 ± 1.28	60.27 ± 1.66	65.96 ± 1.64
**14c**	14.85 ± 1.36	22.56 ± 1.79	26.29 ± 1.23	39.48 ± 1.61
**14d**	39.91 ± 1.75	41.82 ± 1.49	44.96 ± 1.37	55.44 ± 1.33
**14e**	17.89 ± 1.23	28.18 ± 1.27	30.87 ± 1.98	34.89 ± 1.72
**14f**	9.05 ± 1.63	10.15 ± 1.83	16.19 ± 1.21	22.93 ± 1.62
**14g**	8.39 ± 1.14	11.87 ± 1.23	14.08 ± 2.01	26.55 ± 2.13
**14h**	57.93 ± 1.36	67.35 ± 1.35	72.00 ± 1.82	85.99 ± 2.17
**14i**	39.91 ± 1.87	41.82 ± 1.09	44.96 ± 1.66	55.44 ± 1.55
**15a**	9.36 ± 1.25	14.15 ± 1.61	20.39 ± 1.92	24.77 ± 2.05
**15b**	23.60 ± 1.36	32.73 ± 1.13	39.23 ± 1.29	47.48
**Negative control**	0	0	0	0
**Ascorbic acid**	94.37 ± 1.74	97.45 ± 1.32	98.78 ± 0.94	99.67 ± 0.28

^a^ Results are the mean of three independent experiments. Data = mean ± SD.

**Table 3:** Antiproliferative activity of the newly synthesized compounds against human carcinoma cell lines and normal skin fibroblast cells (BJ-1) at 100 µg/ml.

Compd. No.	Growth Inhibition (%)				Growth Inhibition (%)				
	HCT-116	HepG-2	MCF-7	BJ-1	Compd. No.	HCT-116	HepG-2	MCF-7	BJ-1
**11a**	96.2	-	94.3	6.2	**13g**	92.4	81.9	91.6	13.0
**11b**	94.9	-	92.3	10.1	**13h**	91.3	79.5	93.4	14.1
**11c**	30.2	-	0	45.2	**13i**	97.7	96.8	97.8	69.4
**12a**	96.7	-	93.6	9.1	**14a**	91.2	-	90.4	14.2
**12b**	95.1	-	97.4	5.0	**14b**	84.6	-	80.3	8.1
**12c**	12.4	-	6.3	62.3	**14c**	91.8	-	92.3	13.6
**13a**	96.9	89.3	96.4	11.6	**14d**	96.2	-	94	9.5
**13b**	94.5	81.4	95.4	10.4	**14e**	35.1	-	85.3	16.6
**13c**	97.9	90.9	97.8	5.1	**14f**	91.4	-	89.3	14.5
**13d**	94.6	82.7	96.4	7.0	**14g**	92.1	-	90.6	16.0
**13e**	90.4	79.8	96.4	10.3	**14h**	96.2	-	94	86.5
**13f**	86.5	72.8	79.7	16.4	**14i**	35.1	-	52.3	78.6
**Doxorubicin**	100	100	96.8	0		100	100	96.8	0

**Table 4:** IC_50_ values of spiro pyrazole-oxindole congeners against human cancer cell lines.

Compd. No.	IC_50_ug/ml		
	HCT-116	HepG-2	MCF-7
**11a**	5.7	-	21.1
**11b**	16.4	-	19.4
**12a**	5.8	-	20.2
**12b**	7.9	-	16.7
**13a**	31.3	60.6	32.4
**13b**	21.3	48.0	24.0
**13c**	14.7	27.0	24.1
**13d**	26.9	60.9	25.4
**13e**	35.4	67.1	31.9
**13f**	28.4	83.6	32.1
**13g**	35.3	72.2	43.0
**13h**	20.5	19.2	5.8
**14a**	36.2	-	32.3
**14b**	40.1	-	36.3
**14c**	40.5	-	32.5
**14d**	30.7	-	31.6
**14e**	-	-	54.0
**14f**	35.8	-	31.2
**14g**	38.0	-	37.4
**Doxorubicin**	26.1	21.6	37.6

All IC_50_ values were calculated for the timepoint 48 h post-treatment.

**Table 5:** Effect of analogues 11a, 11b, 12a, 12b and 13c on cleaved caspase-3 levels, and expression levels of Bcl-2 and Bax in MCF-7 cancer cells treated with the compounds at their IC_50_ concentrations.

Compounds	Caspase-3 Activity %	Bcl-2 (ng/50 mg Protein)	p53 (Pg/50 mg Protein)	Bax (pg/50 mg Protein)
**Control (MCF-7)**	10.2 ± 2.84	25.13 ± 3.45	4.18 ± 0.58	39.56 ± 4.56
**11a**	16.67 ± 0.67	12.3 ± 2.26	14.45 ± 0.82	122.34 ± 3.45
**11b**	16.19 ± 0.79	16.18 ± 0.78	5.85 ± 0.75	109.35 ± 4.05
**12a**	14.18 ± 0.74	13.09 ± 2.27	13.31 ± 1.08	113.09 ± 1.98
**12b**	47.25 ± 1.89	12.87 ± 1.84	29.58 ± 2.53	167.07 ± 4.83
**13c**	39.07 ± 4.97	44.5 ± 4.56	7.34 ± 1.5	88.34 ± 3.79

Data = mean ± SD.

**Table 6:** Effect of compounds 11a, 11b, 12a, 12b and 13c on active caspase-3 levels, and expression levels of Bcl-2 and Bax in HCT-116 cancer cells treated with the compounds at their IC_50_ concentrations.

Compounds	Caspase-3 Activity %	Bcl-2 (ng/50 mg Protein)	p53 (pg/50 mg Protein)	Bax (pg/50 mg Protein)
**Control (HCT-116)**	13.60 ± 2.45	23.56 ± 3.56	1.68 ± 0.06	118.54 ± 0.83
**11a**	19.12 ± 0.89	8.2 ± 1.26	16.2 ± 0.56	159.26 ± 0.96
**11b**	14.04 ± 0.54	7.25 ± 1.45	3.25 ± 0.15	119.14 ± 1.65
**12a**	17.43 ± 0.58	21.13 ± 1.45	23.11 ± 1.22	124.56 ± 0.95
**12b**	33.12 ± 1.37	18.87 ± 1.84	48.07 ± 1.94	191.07 ± 1.95
**13c**	47.32 ± 6.32	57.45 ± 6.45	2.34 ± 0.95	123.45 ±7.45

Data = mean ± SD.
